# Nasal delivery of neurotherapeutics *via* nanocarriers: Facets, aspects, and prospects

**DOI:** 10.3389/fphar.2022.979682

**Published:** 2022-09-13

**Authors:** Amarjitsing Rajput, Prashant Pingale, Vividha Dhapte-Pawar

**Affiliations:** ^1^ Department of Pharmaceutics, Poona College of Pharmacy, Bharti Vidyapeeth Deemed University, Pune, India; ^2^ Department of Pharmaceutics, GES’s Sir Dr. M. S. Gosavi College of Pharmaceutical Education and Research, Nashik, India

**Keywords:** intranasal formulations, neurotherapeutics, Alzheimer’s disease, nanocarrier, brain-targeted drug delivery

## Abstract

Alzheimer’s disease (AD) is one of the neurological ailments which continue to represent a major public health challenge, owing to increased life expectancy and aging population. Progressive memory loss and decrease in cognitive behavior, owing to irreversible destruction of neurons along with expensive therapeutic interventions, call for an effective, alternate, yet affordable treatment for Alzheimer’s disease. Safe and effective delivery of neurotherapeutics in Alzheimer’s like central nervous system (CNS) disorders still remains elusive despite the major advances in both neuroscience and drug delivery research. The blood–brain barrier (BBB) with its tight endothelial cell layer surrounded by astrocyte foot processes poses as a major barrier for the entry of drugs into the brain. Nasal drug delivery has emerged as a reliable method to bypass this blood–brain barrier and deliver a wide range of neurotherapeutic agents to the brain effectively. This nasal route comprises the olfactory or trigeminal nerves originating from the brain and terminating into the nasal cavity at the respiratory epithelium or olfactory neuroepithelium. They represent the most direct method of noninvasive entry into the brain, opening the most suitable therapeutic avenue for treatment of neurological diseases. Also, drugs loaded into nanocarriers can have better interaction with the mucosa that assists in the direct brain delivery of active molecules bypassing the BBB and achieving rapid cerebrospinal fluid levels. Lipid particulate systems, emulsion-based systems, vesicular drug delivery systems, and other nanocarriers have evolved as promising drug delivery approaches for the effective brain delivery of anti-Alzheimer’s drugs with improved permeability and bioavailability *via* the nasal route. Charge, size, nature of neurotherapeutics, and formulation excipients influence the effective and targeted drug delivery using nanocarriers *via* the nasal route. This article elaborates on the recent advances in nanocarrier-based nasal drug delivery systems for the direct and effective brain delivery of the neurotherapeutic molecules. Additionally, we have attempted to highlight various experimental strategies, underlying mechanisms in the pathogenesis and therapy of central nervous system diseases, computational approaches, and clinical investigations pursued so far to attain and enhance the direct delivery of therapeutic agents to the brain *via* the nose-to-brain route, using nanocarriers.

## 1 Alzheimer’s disease

### 1.1 Introduction

Alzheimer’s disease (AD) is considered a fatal irreversible neurodegenerative disorder mainly affecting the brain (Mathys et al., 2019). AD shows signs of early stages such as dementia, which steadily worsen, impacting daily activities and relations (Duan et al., 2018). The presence of plaques and tangles in the brain suppresses the neuronal transmission, an essential hallmark of AD (Pathak et al., 2021; NIH, 2022). Lifestyle parameters do not directly impact the pathological state of AD but can still result in positive outcomes in persons with AD (Scheltens et al., 2021).

Across the globe, 50 million people (approximate) more than 60 years of age are suffering from dementia and 50–70% of population from AD (Niu et al., 2017; Collaborators, 2019). The number of people with AD will be more than 115 million by 2050 (Dementia, 2020; Scheltens et al., 2021). In the United States, around 3% population aging between 65 and 74, 17% population aging between 75 and 84, and 32% population aging 85 and above are suffering from AD (Hebert et al., 2013; Ageing, 2021). The cases of AD double every 10 years in population aging more than 60 (Eratne et al., 2018). It kills more people than breast and prostate cancer do. During the COVID-19 pandemic, AD caused 16% of deaths. AD and other dementias incurred the United States to spend $355 billion in 2021, and this cost could climb to more than $1.1 trillion by 2050. More than six million adults in the United States of America (USA) suffer from AD, and it will reach up to 14 million by 2050 (Dementia, 2020; Figures, 2021).

### 1.2 Pathophysiology and potential target

Diverse drug molecules are developed for treatment of various CNS ailments like the following: treatment of epilepsy: amiloride and oxcarbazepine.

Treatment of schizophrenia: olanzapine, risperidone, and quetiapines.

Treatment of Alzheimer’s disease: rivastigmine and memantine.

Treatment of Parkinson’s disease: resveratrol and selegiline.

Treatment of bacterial meningitis: chloramphenicol.

These currently available/approved drugs have restricted access to the brain due to the presence of blood–brain barrier (BBB), thus creating a significant challenge in attaining therapeutic effectiveness. In addition, availability of minute pores and drug efflux transporter (P-glycoproteins) systems in the BBB leads to a decrease in the therapeutic concentration of compounds in the central nervous system (CNS) (Gao, 2016). The saturation of vehicles present on the edge of the BBB can result in lower drug amount in the brain (Summerfield et al., 2016). For example, memantine showed lower concentration in different parts of the brain in spite of excellent oral bioavailability (Beconi et al., 2011). The effect may be due to the saturation of in-flux transporters, that is, organic cationic transporters (OCTN 1–3) on the BBB (Mehta et al., 2013). Similarly, donepezil (Al Harthi et al., 2019) and galantamine exhibited low levels in the brain, irrespective of high oral bioavailability (Misra et al., 2016). Rivastigmine is linked with the issues of a shorter half-life due to high first-pass effect (Wavikar et al., 2017). These marketed compounds are used in the management of AD and thus showed poor brain penetration that decreased their therapeutic potential. Other challenges with these drugs are nephrotoxicity (Horikawa et al., 2013; Tsukamoto et al., 2013), hepatotoxicity (Ferrara et al., 2008; Hirono et al., 2018), and gastrointestinal adverse effects (Leonard et al., 2007). Hence, intranasal drug delivery would help in the effective delivery of neuroactives into the CNS, with minimal drug exposure to peripheral body parts. This may further add benefits like enhanced patient compliance, increased therapeutic effectiveness, faster onset of action, and prevention of the first-pass effect (Agrawal et al., 2018). Thus, to overcome the challenges of current treatment methods and attain desired therapeutic concentration of actives into the brain, intranasal delivery serves as a promising route.

The most important causative parameter and efficient strategy in AD management is still not known, although there is sufficient literature works on the basic biological and clinical neuropathology of AD (Guo et al., 2020). The chances of developing AD rely on various factors like genetic, non-genetic, and causative factors (Sharma et al., 2020) like the aggregation of Aβ peptides and neurofibrillary tangles (NFT). Another hypothesis suggested that dysfunction or enlargement in the perivascular spaces inside the brain develops and results into neurological diseases such as AD, dementia, and cerebral amyloid angiopathy (Plog and Nedergaard, 2018; Gouveia-Freitas and Bastos-Leite, 2021).

In the last five decades, numerous novel compounds and treatment methods are clinically studied in the management of CNS ailments with a huge investment by pharmaceutical industries. However, merely 8% of these antipsychiatric products reached the market (Mohs and Greig, 2017). Most of these treatments for neurodegenerative diseases simply reduced the disease’s progression rather than curing it entirely. Majority of hydrophilic compounds are unable to cross the BBB, owing to the presence of the efflux transporters like P-glycoprotein (P-gp) and other multi-drug-related protein transporters (Matsumoto et al., 2017; Patel et al., 2018). Due to their poor ability to cross the BBB, high first-pass effect, and frequent off-site targeting (Goel et al., 2022), and prospective peptide-based (Ayyalasomayajula and Suresh, 2018; Trapani et al., 2018) or gene delivery (Malhotra et al., 2013)-based therapies continue to be in the translational phase (Goel, et al., 2022).

Currently, six drugs including the recently investigated monoclonal antibody Aduhelm™ (Aducanumab) used in the management of AD are approved by the US Food and Drug administration (FDA) (Grill and Cummings, 2010; Alzheimer’s, 2021). These compounds have resulted in crucial and helpful symptomatic advantages but have limited or no effect on the underlying biology/progression of AD. The acetylcholinesterase inhibitors (AChEIs) inhibit the breakdown of acetylcholine and maintain its level in the brain (example: donepezil). Another class of drug approved by the USFDA includes memantine, which acts by controlling the excitatory glutamatergic role and enhances cognition in moderate to severe AD (Tariot et al., 2004; van Dyck et al., 2007). The combination therapy of these classes of drugs resulted in enhanced symptomatic and sustained effects (Atri et al., 2008; Lopez et al., 2009). However, current therapies suffer from many side effects and limitations.

1) Genomics: genomics opens the doors for efficient delivery of neurotherapeutics, but it requires significant individualization depending on the condition. The formulation of molecules targeting individualized therapy is time-consuming and costly. Each molecule needs to meet regulatory guidelines involving studies in large population (Horrobin, 2001).
2) Specificity: the major challenges associated with neurotherapeutics start with complex, anatomically specific, and temporally intense behavior of the target organ, that is, brain functioning. (Schreiber, 2000).
3) Animal models: the selection of the animal model for CNS disease is critical as it is very essential to understand their validities such as pace, and predictive and constructive validities along with abnormalities in selected animal models (Willner, 1991).
4) Trial conduct: conducting the clinical trials of neurotherapeutic formulations in resemblance to other trails is challenging. Clinical trials are very common to the project management tasks, but neurotherapeutics trials consist of unusually intense challenges (Rowland, 1995).

Hence, there is a necessity to explore nanoplatforms to tackle these problems.

### 1.3 Targeting approaches in the management of Alzheimer’s disease

The various drug delivery mechanisms utilized to disrupt or overcome the BBB and increase drug molecule transport across this barrier to the CNS have been investigated in the management of AD. These approaches are classified into invasive, noninvasive, and current BBB disruption therapies (Khan et al., 2017). Invasive approaches include convection-improved delivery, focused ultrasound-improved delivery, disruption of the BBB by chemicals, craniotomy-based drug delivery, polymeric wafers, and chip technology. Craniotomy drug delivery involves intra-cerebral/ventricular injection into the ventricles or subarachnoidal parts of the brain. However, higher doses are required for the desired therapeutic effect. Noninvasive approaches are composed of cell-based therapy, efflux pump inhibition, nanocarrier-loaded drug delivery, intranasal delivery, and prodrug approach. The prodrug approach aids in improving brain delivery by modulating the lipophilicity and permeability of the active molecules. 1, 4-Dihydrotrigonellyl chemical attached to Tyr-D-Ala-Gly-Phe-D-Leu, a prodrug of enkephalins, improved its lipophilicity and permeability across the BBB and peptidase enzymes, respectively, and later delinked the spacer for drug release at the desirable site. The current BBB disruption therapies in brain-targeted drug delivery contain facial intradermal injection, antibody-mediated drug delivery, mfsd2a-based drug delivery, and laser light technology (Khan et al., 2017). A novel mfsd2a-based drug delivery strategy helped in the treatment of AIDS-induced encephalopathy wherein reverse transcription of HIV in the brain was inhibited by Efavirenz-loaded nanocarriers. This mechanism was associated with the transport of specific lysophosphatidyl-choline (LPC) derivatives (Vyas et al., 2015). Laser light induces defects in endothelial cell membranes and makes them leaky for transporting 5-amino levulinic acid (5-ALA) to parenchymal tissues, specifically as a part of effective glioma treatment (Fernandez and Pozo, 2012).

### 1.4 Intranasal pathway for brain targeting

**FIGURE 1:**
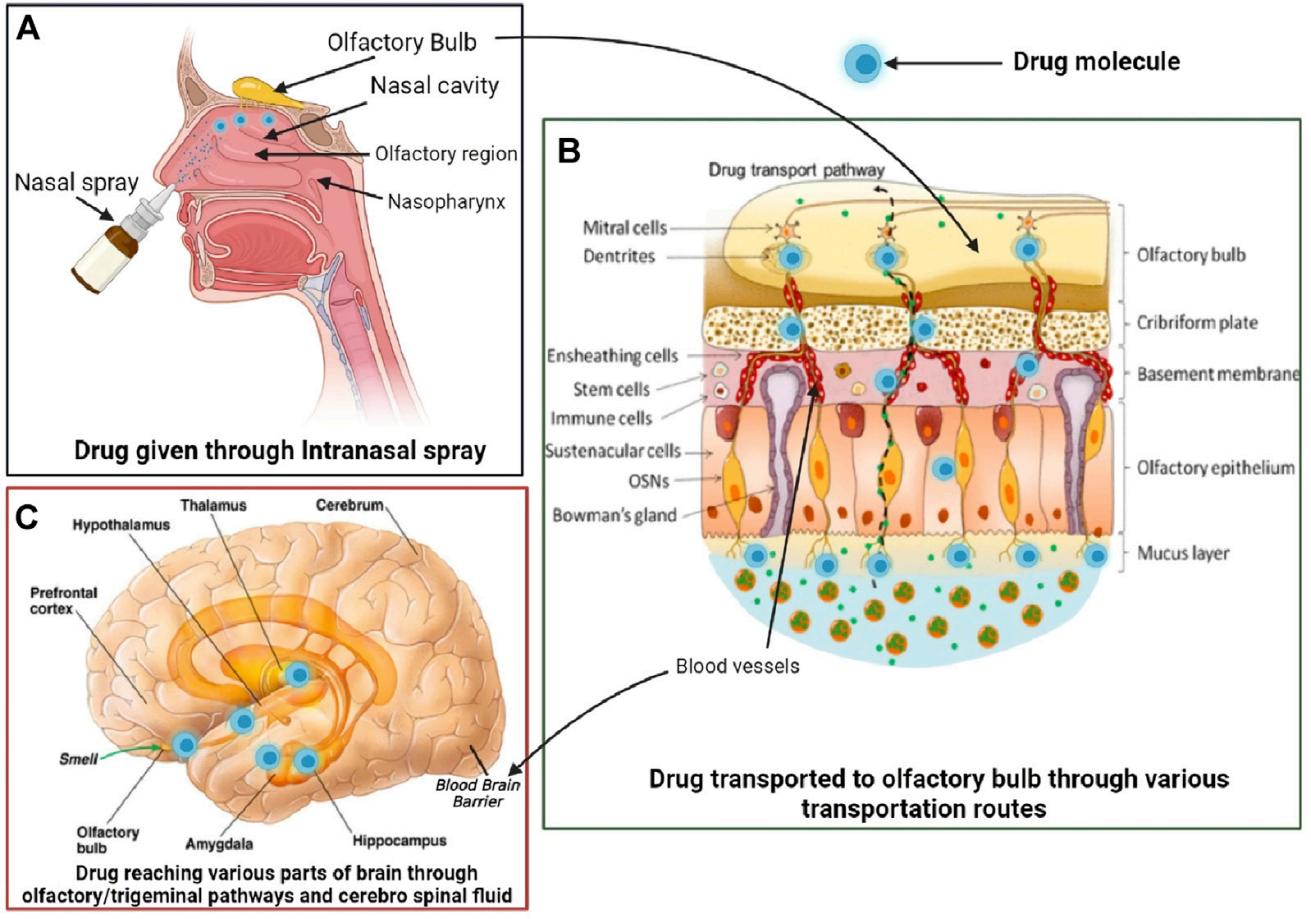
Intranasal drug delivery mechanisms for brain targeting.

The intranasal pathway for brain targeting is useful in the management of different neurodegenerative diseases like Alzheimer’s, schizophrenia, Parkinson’s, epilepsy, meningitis, and brain tumor. The common intranasal transport pathway for brain targeting includes passive partitioning, carrier-mediated transport, and paracellular pathway (Abbott et al., 2006; Konsman et al., 2007). It also consists of intracellular or neuronal translocation (Crowe et al., 2018), extracellular (Dhuria et al., 2010), and perineural or perivascular spaces of blood (Lochhead and Davis, 2019) and also serves as an important pathway for intranasal delivery, as depicted in Figure 1. As the olfactory pathway is the shortest route between the nasal cavity and the brain and is known to bypass the blood–brain barrier (BBB), intranasal delivery is apt for immediate drug action. Also, olfactory physiology offers advantages such as deep vascularization, porous endothelial membrane, and large surface area for effective brain delivery (Mustafa et al., 2016).

## 2 Nanocarrier-based systems for management of Alzheimer’s disease (AD)

### 2.1 Role and advantages of nanocarriers used in AD

The existence of the BBB limits therapeutic development in AD. More than 95% of small biomolecular medicines and all of the large molecular pharmaceuticals fail to pass across the BBB. The difficulty of transporting medications over the BBB is one of the most significant concerns and obstacles in the development of novel therapies and therapeutic measures for AD (Pardridge, 2009). The negative therapeutic impact of pharmaceuticals can be caused by their physicochemical features such as low bioavailability, hydrophobicity or lipophilicity, ionization, extensive metabolism, large molecular weight, and likely complications. When traditional medication dosage forms are employed for brain drug delivery, most of these issues arise. In terms of high precision, excellent durability, high drug-loading factor, ability to transport hydrophilic and hydrophobic drugs, and feasibility of different drug administration routes, the lipid nanoparticle-based drug delivery system has various advantages over other available drug delivery systems. Therapeutic options are limited mainly due to pharmaceuticals’ inability to pass the BBB or their relatively poor solubility. Many lipidic nanoparticulate approaches such as solid lipid nanoparticles, nanostructured lipid carrier, microemulsions, and liposomes are fabricated to cross the BBB (Roney et al., 2005). Lipid nanoparticles have several advantages for CNS medication delivery as listed:

• With significant advantage in crossing the lipophilic BBB, particle manufacturing procedures for lipid nanoparticles are easy to scale up. Lipidic nanoparticles easily penetrate the BBB.
• Pharmaceuticals are preserved from chemical and enzymatic breakdown by lipidic nanoparticles. They can also help nullify the negative effects of some pharmaceuticals.
• Diverse active molecules have been successfully targeted to brain tissue using nanoparticles (antibiotics, protein, cytostatic, peptides, and nucleic acids).
• Without surface modification or functionalization, lipidic nanoparticle medicines can be transported directly to the CNS (which may affect the efficacy). This technique can be employed in a variety of ways, including intradermal, oral, nasal, and parenteral delivery.
• Nanoparticles, having compact morphology, easily penetrate through capillaries and are taken up by biomolecular cells, allowing for an effective medicine at the targeted areas in the body.
• Biodegradable polymers used in the formulation of nanoparticles provide prolonged medication release at the targeted location for days or even weeks, following injection (Kamaly et al., 2016).

### 2.2 Lipid particulate system

The lipid particulate system for nose-to-brain delivery is one of the most commonly used approaches for improved drug delivery to the brain. As these systems are amphiphilic, they can deliver both hydrophobic and hydrophilic neuroactive molecules. Biocompatible lipid particulate systems are made using biodegradable lipids which make them more biocompatible physiologically with less probability for toxicity. Types of lipids such as 1,2-distearoyl-sn-glycero-3-phosphocholine (DSPC), poloxamers, cholesterol, polyethylene glycol (PEG), capmul MCM, precirol, lecithin, and compritol 888 ATO along with the size and charge on lipid excipients have a profound impact on the nose-to-brain delivery of neurotherapeutic drugs (Lee and Minko, 2021). Positively charged lipid carriers are found to have an effective intranasal delivery than neutral or anionic charged lipid carriers as they are easily attracted by the anionic endothelial cells of the brain *via* absorptive mediated transport. Nasal epithelium possesses negative charge, and hence, cationic lipid nanocarriers can demonstrate better interaction and bioadhesion during intranasal delivery. Few reports have shown an effective nose-to-brain delivery by neutral- and anionic-charged lipid nanocarriers *via* endocytic or transcellular mechanisms (Gabal et al., 2014).

#### 2.2.1 Solid lipid nanoparticles (SLNs)

SLNs are thought to be desirable colloidal drug delivery methods for brain targeting. The ability of these formulations to cross the BBB, which enables the medicine to enter into the CNS in useful quantities, is one of the most intriguing problems they face. High drug concentrations are required for neuroprotection, a situation that is challenging to attain due to efflux processes that move treatments from the brain to circulation and, most importantly, because medications are unable to penetrate the BBB. SLNs have emerged as a promising candidate in terms of safety, stability, low toxicity, large scale production, and drug loading. Many researchers attempted to address this problem in recent years using medicinal chemistry-based strategies and modern drug delivery systems (Sozio et al., 2010; Cacciatore et al., 2012).

It was discovered in one study involving the encapsulation of piperine, a natural alkaloid with a polyene bond system which provides antioxidant property and a tertiary nitrogen for mimicking the acetylcholine structure. Its administration to the brain is difficult to achieve as it undergoes extensive metabolism in the liver, carries risk of polymerization, and poor water solubility. To address this, SLNs were made with glycerol monostearte and various coatings, like polysorbate-80, for effective brain targeting. *In vitro* and *in vivo* testing revealed that SLNs of piperine coated with polysorbate-80 reduced superoxide dismutase levels, reduced cholinergic degradation, caused minimum immobilization in the animal, and reduced the presence of amyloid plaques (Yusuf et al., 2013).

A natural flavonoid quercetin was formulated into the SLN as a potential medicinal moiety for the treatment of AD. It was discovered that treating aluminum chloride-treated rats with quercetin-loaded SLN revealed decreased neurodegenerative effects and, more specifically, that the antioxidant capacity of quercetin was greatly enhanced by SLN formulation (Dhawan et al., 2011).

#### 2.2.2 Nanostructured lipid carrier (NLC)

The liquid lipid causes disarray in the lipid matrix of the NLC, allowing for increased encapsulation efficiency and minimal ejection of the encapsulated medication during storage. As a result, the current research focuses on the NLC as a nanocarrier for brain targeting. NLC has benefits over other nanosystems for nasal medication delivery since it is made from biocompatible and biodegradable materials such as physiological lipids and other excipients. Furthermore, the NLC shields medicines from enzymatic breakdown, extends nasal cavity residence duration, and enhances bioavailability. Furthermore, the NLC with desirable properties for nose-to-brain delivery can be produced (Jojo et al., 2019; Cunha et al., 2021).

Optimized pioglitazone-loaded NLCs are used for nose-to-brain administration in one of the reported studies. Pioglitazone is an antidiabetic medication that might be used to address numerous targets in AD. The formulation was found to be safe for nasal administration in an *in vitro* nasal ciliotoxicity investigation. After nasal injection, *in vivo* biodistribution showed that pioglitazone-loaded NLC delivered more pioglitazone to the brain, suggesting the potential of NLC as an effective pioglitazone carrier in AD therapy (Chintamaneni et al., 2017).

#### 2.2.3 Lipid drug conjugates (LDCs)

**FIGURE 2:**
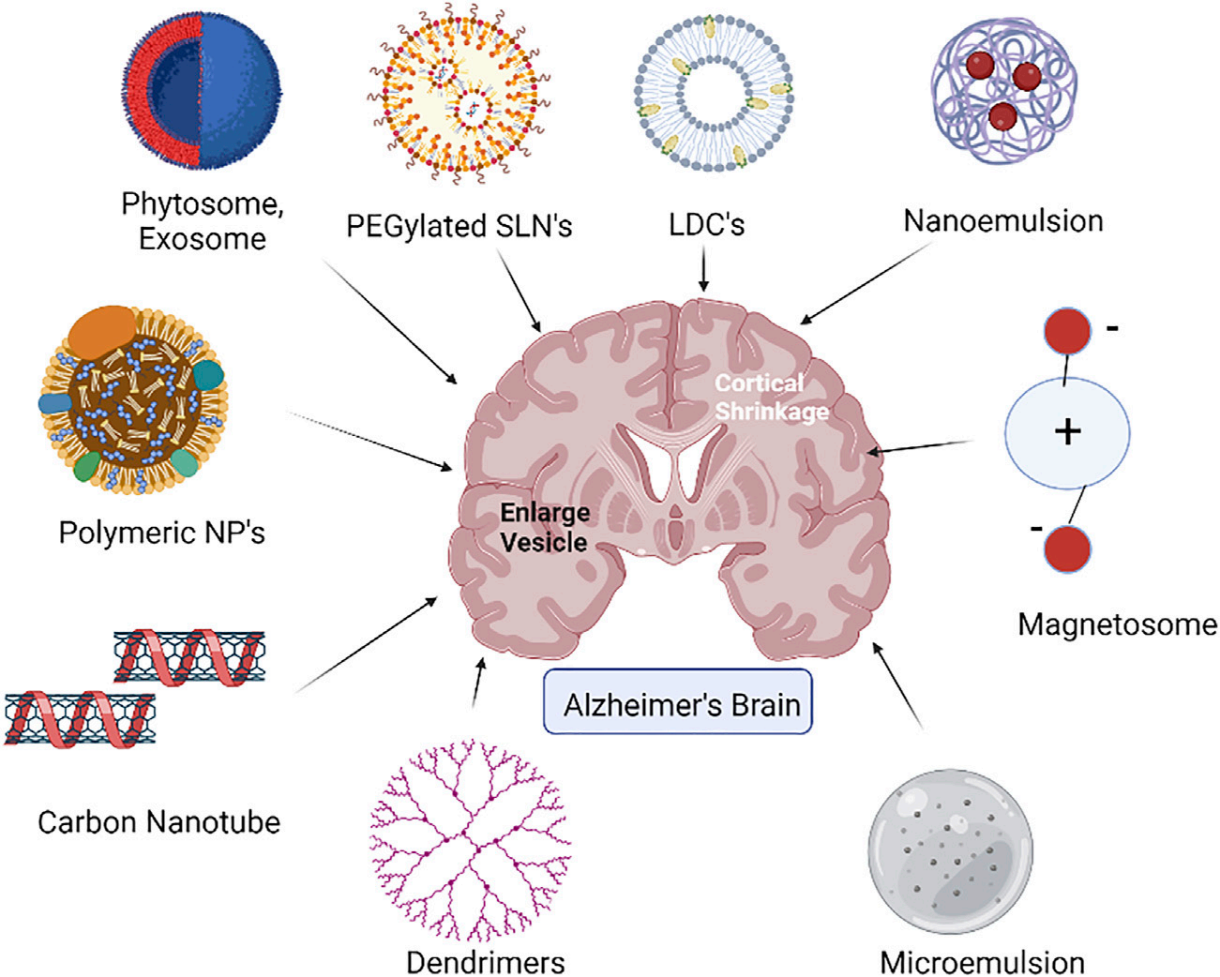
Diverse nanocarriers for the effective nose-to-brain delivery in Alzheimer’s treatment.

LDCs as a drug delivery device aid in improving the medication’s lipophilic properties. Due to its particle size and surface characteristics, LDCs are vital for brain-targeted drug delivery (Figure 2). As a result, LDCs are formed as nano-LDCs using high-speed and high-pressure homogenization procedures to produce particles in a particle size range of 100–200 nm that can pass through the GI tract and the BBB. Positive allosteric modulators (PAMs) of M1 muscarinic receptors have no agonist action, but when attached, they increase the affinity of the orthosteric ligand, ACh. The bioavailability of these PAMs in the brain is one of their primary drawbacks. The bioavailability of PAMs of the M1 receptor has been found to be improved by surface-modified nano-LDCs (Chintamaneni et al., 2017). When used in conjunction with AChE inhibitors, they are predicted to improve the effectiveness while lowering therapeutic dose and adverse effects. PAMs can be made as LDC nanoparticles coated with polysorbate-80 to improve oral bioavailability and its penetration to the CNS while simultaneously lowering clearance.

#### 2.2.4 Microemulsion (ME)

MEs are pseudoternary compounds that are thermodynamically stable, are isotropic in nature, and have low-viscosity physicochemical features. ME droplet sizes range from 10 to 140 nm. Due to less particle size, they are both optically clear and thermodynamically stable (Figure 2). MEs have a number of benefits, including the ability to carry high amounts of lipophilic and hydrophilic medicines, as well as focused and regulated drug administration, and are thus being studied extensively for intranasal delivery in brain targeting. ME components such as oils, surfactants, and cosurfactants generally serve as absorption enhancers, P-glycoprotein (P-gp) inhibitors, and improve drug uptake in the brain in order to cross the BBB. To increase brain delivery, functional MEs might be created using such functional additions (Kamaly et al., 2016). Ibuprofen was repurposed using the ME strategy in the therapy of AD and was found effective in brain targeting *via* the intranasal route as compared to oral and intravenous routes (Wen et al., 2021). Oils such as oleic acid along with Tween 20, ethanol, and propylene glycol as surfactants and cosurfactants in ibuprofen-repurposed o/w ME helped as penetration enhancers on the tight junctions and enzyme inhibitors in the nasal cavity to overcome enzymatic degradation in the nasal cavity.

### 2.3 Emulsion-based systems

#### 2.3.1 Nanoemulsion (NE)

Thermodynamic stability and enhanced pharmaceutical design make these nanosized emulsions (NEs) attractive platforms for successful transport of bioactive molecules across the BBB. NE as a viable medication delivery strategy for the therapy of neuronic illnesses is enabled by innovative surfactants and cosurfactants. Their tiny particle size (50–500 nm) allows for homogeneous dispersion of medicines and a larger payload for site-specific delivery (Figure 2). Intranasal medication delivery with a memantine-embedded NE has been described. This method circumvented the BBB and successfully delivered memantine in the treatment of AD. In simulated nasal fluid, the NE had a mean globular size of approximately 11 nm and 80% drug release. Intranasal injection of a memantine-loaded NE resulted in increased antioxidant effectiveness and improved cellular absorption in experimental rats’ brain cells. The oil by water (o/w) donepezil hydrochloride-loaded NE was made with 10% labrasol and glycerol. The AD-infected brain cells were influenced by nanoscale, homogeneous, and stable particles of NE. The nasal route was used to study the cytotoxicity in Sprague–Dawley rats, which indicated dose-dependent effectiveness without disrupting the cell shape. The antioxidant and radical scavenging effectiveness of donepezil hydrochloride NE demonstrated great potential for treating neurological disorders. The scintigram results determined the maximal cellular absorption of donepezil hydrochloride by brain cells (Cunha et al., 2021).

### 2.4 Vesicular drug delivery systems

#### 2.4.1 Liposomes

Liposomes are self-aggregated, lipidic nanomolecules that combine and deliver lipophilic, hydrophilic, nucleic acid components and proteins to the CNS. Liposomes with lipophilic properties aid medication transportation through the endothelium cells inside the brain and allow excellent absorption in the brain (Vieira and Gamarra, 2016; Hong et al., 2019; Pathak et al., 2021). The two most common routes for drug release from liposomes are drug diffusion and liposomal endocytosis. The structure and lipid composition of vesicles, on the other hand, have an effect on their transportation *via* circulation and cellular absorption in the brain cells. The vesicular morphology of liposomes helps in delivery of both hydrophilic and hydrophobic neuroactives. The liposome research has shown that they have a wide range of pharmacological activities like anti-ischemic, neuroprotective, antiepileptic, and antibacterial properties. These formulations have been investigated as cargos for therapeutic import across the BBB by active targeting (Pathak et al., 2021). One of the research projects includes the development of curcumin-loaded liposomes for the effective treatment of AD. Curcumin-loaded liposomes illustrated antiamyloid properties. The findings showed that amyloid poisons generated in the brain endothelium area possess neuroprotective properties. Curcumin’s phenolic group binds to amyloid proteins, reducing the deposition of plaque and facilitating an aggregation-free environment surrounding the brain cells (Behl et al., 1994).

#### 2.4.1 Niosomes

Niosomes are made up of nonionic surfactants that self-assemble in an aqueous system, making them more versatile and durable than liposomes while also lowering the drug permeability. One of the experiments undertaken by researchers focused on the possibility of unique dual drug-loaded niosomes for the nasopharyngeal transport of rivastigmine and N-acetyl cysteine to the CNS. The dual drug-loaded niosomes had a size distribution of 162 nm with an encapsulation efficiency of 97% for rivastigmine and 85% for N-acetyl cysteine. The dual drug-loaded niosomes’ drug release pattern sustained up to 2 days. Free drug formulations had a greater combinatorial impact than inhibition experiments for the enzymes acetyl cholinesterase and DPPH (1, 1-diphenyl-2-picrylhydrazyl). After a 2-day nasal permeation, the niosomes’ efficacy and biocompatibility were established. *In vivo* pharmacokinetic and organ biodistribution experiments have revealed that the niosomes had a superior pharmacological profile and a wider distribution in the brain than other organs, indicating targeted nose-to-brain delivery (Saraswathi et al., 2021).

#### 2.4.2 Phytosomes

Phytosomes are made up of individual components from herbal extracts linked to phosphatidylcholine (Figure 2). In India, *Geophila repens* is a climbing plant found to have anticholinesterase qualities and hence is used for improving memory performance. The antioxidant capabilities of *Geophila repens* leaves were examined and found to be significant, presumably attributed to the prevalence of triterpenoids, phenolic chemicals, and flavonoids. Systemic absorption of phytochemical compounds *via* the BBB is challenging for developing phytoformulations for AD therapy. To overcome these concerns, the *Geophila repens* phytosome-loaded intranasal gel was prepared with better nasal–brain penetration capabilities. In this experiment, transcutol was used as a permeation enhancer, while hydroxypropyl methyl cellulose (HPMC) was used as a gelling agent. During *ex vivo* studies, this gel exhibited significant penetration across the nasal mucosa without any irritation. Furthermore, it had a similar proportion of acetyl cholinesterase inhibition to donepezil (97%) but was higher than 20% from normal *Geophila repens* leaves’ gel and accelerated angiogenesis, indicating that it has the potential to change the cognitive behavior of Alzheimer’s condition in patients (Rajamma et al., 2022).

#### 2.4.3 Exosomes

Exosomes are nano-sized extracellular vesicles produced by endosomes and released by practically all cell types. Exosomes are abundant in biological fluids and CNS tissues (Figure 2). Their contents change during illnesses, making them an appealing target for innovative diagnostic techniques. Exosomes have been demonstrated to transfer toxic amyloid-beta and hyperphosphorylated tau across cells, thus triggering apoptosis and contributing to neuronal death. On the other hand, exosomes appear to have the capacity to lower amyloid-beta levels in the brain *via* microglial absorption and transport neuroprotective chemicals across cells. Exosomes are particularly fascinating from the point of creating innovative treatment methods because of these characteristics, among many others. Exosomes generated from the CNS are also prevalent in physiological fluids, making them a promising target for biomarker development (Zhang et al., 2019). Exosomes have been implicated in AD since their discovery, and various investigations have established their multiple functions in the disease. Exosomes appear to be valuable in a variety of ways in the diagnosis and treatment of AD, such as reducing the extracellular amyloid load or functioning as early indicators of the illness (Cai et al., 2018; Soliman and Ghonaim, 2021).

#### 2.4.4 Magnetosomes

Magnetosomes have attracted a lot of attention in recent years, and they are being employed for both diagnostic and therapeutic applications (Gorobets et al., 2017). With their neutral nature and low cytotoxicity, magnetosomes may be effective in brain disorders like AD (Figure 2). One of the investigations reveals that magnetic nanoparticles can permeate the normal BBB when a mouse model is exposed to an external magnetic field. An electromagnetic controller was developed to strategically direct magnetite-containing drugs. These nanoparticles were able to cross the BBB after being exposed to external electromagnetic fields of around 28 mT (0.43 T/m). Magnetic nanoparticle absorption and transportation rates in the brain were also dramatically boosted by a pulsed magnetic field. The localization measured by fluorescent magnetic nanoparticles demonstrated the viability of magnetic nanocontainers as a useful targeted device for AD diagnosis and treatment (Kong et al., 2012).

### 2.5 Other nanocarriers

#### 2.5.1 Dendrimers

Dendrimers are the most symmetric, hyperbranched, and homogeneous nanosized carriers that can deliver water-soluble or -insoluble, high- or low-molecular weight molecules either by passive or active mechanisms by intravenous, intraperitoneal, or intranasal routes of administration. *In situ* mucoadhesive gels showed enhanced radioactivity in the brain post intranasal delivery of aqueous, radio-labeled siRNA-dendrimer complex (dendriplexes), owing to the diffusion mechanism (Perez et al., 2012). Cationic charged surface of dendriplexes improves the association with mucus and, hence, promoted drug delivery to the brain. Polyamidoamine (PAMAM) dendrimers with 15-nm size helped in brain targeting of a poor water insoluble and antipsychotic drug haloperidol by intranasal and intraperitoneal routes of administration (Katare et al., 2015). Intranasal delivery of haloperidol showed better targeting to the brain, especially in the striatum than intraperitoneal delivery and that too at 6–7 times lower dose. Additionally, it could be derived from this study that the intranasal delivery can help in decreasing the CNS side effects by lowering the plasma drug concentration and is used in the management of AD (Aliev et al., 2019) (Figure 2).

#### 2.5.2 Carbon nanotubes

Carbon nanotubes (CNTs) are inorganic materials made of graphite sheet tubes of nanosized dimensions. CNTs can be either single-walled or multi-walled, with open ends or may be closed with fullerene caps. Recently, multi-walled carbon nanotubes (MWCNTs) have been found to exert neuroprotective effects by the modulation of vital neurotrophic factors when delivered *via* the intranasal route (Soligo et al., 2021). Electroconductive MWCNTs have the potential for targeted delivery to various areas in the brain, especially the limbic region, which is the focal point for progression of major neurodegenerative diseases (Figure 2). Thus, MWCNTs can be useful in the therapy of early diabetic encephalopathy, characterized by acute neurodegeneration and poor neurocognitivity (Lohan et al., 2017). CNTs are associated with several serious toxicities such as cellular, respiratory, liver, dermal, subcutaneous, central nervous system, kidney, cardiovascular, and eye toxicities (Mohanta et al., 2019). In case of CNS toxicity, the interaction of CNT with brain cells leads to the release of different mediators/chemicals from microglia and astrocytes that may result in apoptosis, inflammation, and oxidative stress in the brain (Bardi et al., 2013).

#### 2.5.3 Nanomicelles

Nanomicelles are nanosized carriers (particle diameter 10–100 nm) molded by self-association of amphiphilic surfactants or copolymers in aqueous solutions (Bose et al., 2021). It comprises a core (hydrophobic) and a shell (hydrophilic) (Milovanovic et al., 2017). Mixed nanomicelles are more stable and ensure better aqueous solubility and drug-loading efficiency. Stable nanosized mixed lurasidone micelles showed sustained release and better brain targeting of lurasidone hydrochloride by the intranasal route than the intravenous route. The nanosize of lurasidone micelles allowed improved delivery to brain tissue *via* olfactory and trigeminal systems, bypassing the BBB (Pokharkar et al., 2020). PEGylated nanomicelles helped in better uptake by olfactory and trigeminal pathways of cell-penetrating peptides (CPPs) owing to enhanced diffusion across the brain stroma (Kanazawa et al., 2017). Thus, nanomicelles are useful in brain targeting of CPPs, which possess the challenge of limited dose to overcome potential immunogenicity problems.

## 3 Future perspectives in treatment of Alzheimer’s disease

Powder dosage forms and specially designed devices are currently explored for better nasal delivery of vital actives as these approaches can overcome the primary barriers in the olfactory epithelium, namely, the nasal valve and the nasal vestibule. The particle size in the range of 1–10 μm is reported to be suitable for better olfactory deposition (Trevino et al., 2020). Some of these specialized intranasal targeted devices have entered the translational phase of clinical trials for promising delivery of sumatriptan and insulin. An aero pump system (Aero Pump, Germany) working on spring mechanism with an integrated backflow block aids in nose-to-brain delivery of insulin without contamination. The finger actuated, metered nasal dispenser (PharmaSystems, Canada) is a robust, intranasal delivery device for daily administration during the extended period, especially recommended predominantly toward peripheral effects. The semi-disposable, unit-dose, Precision Olfactory Delivery^®^ (Impel Neuropharma, USA) device uses hydrofluoroalkane as a gas propellant to deliver liquids and powders to the olfactory epithelium to ensure consistent dosing with better brain bioavailability. Electronic atomizers generate vortex of nebulized particles to enhance the uptake in the upper nasal cavity with minimum pharyngeal deposition. One such developed device is ViaNase™ (Kurve Technology, Inc. Lynnwood, WA, and USA) for precise dosing and better nose-to-brain delivery of insulin. This device enhances cortical blood flow, vasoreactivity, and cognition.

With more biotechnological and pharmaceutical advances, the avenues in AD therapies are expanding. Mesenchymal stem cells (MSCs) are reported to be valuable in curing the intractable neurodegenerative disorders, owing to their neuroprotective effects exerted by the release of neurotrophic factors (Santamaria et al., 2021). The intranasal route of administration helps circumvent the direct implantation of MSCs and thereby, improve their clinical potential. Intravenous administration of MSC secretome demonstrated cell-mediated neuroreparative effects in APP/PS1 AD mice with temporary memory recovery for a week. Thus, a sustained therapeutic regimen *via* the intranasal route was assessed and found to illustrate persistent memory recovery, with significant decrease of plaques encircled by β-amyloid oligomers. Likewise, researchers are now exploring the nose-to-brain delivery approach for improving feasibility, success rate, and clinical success of simulated nasal casts, gene delivery, and vaccine approaches in the neurotherapies of AD, dementia, or Parkinson’s disease. Clinical studies about intranasal delivery of biomolecules, stem cells, and vaccines are summarized in Table 1, as obtained from the ClinicalTrials.gov database (ClinicalTrials.gov database, 2022). More than 130 trials are in phase 1/2/3. However, there has to be more evidence for the ease of clinical translation and market launch of intranasal neurotherapeutic formulations which could be feasible by the use of nanoformulation and comprehension of mechanistic and immunological aspects.

**TABLE 1:** Clinical status of nanocarriers in Alzheimer’s disease (AD) treatment (Clinical Trials.gov database, 2022).

Drug/treatment	Indication	Formulation	Clinical phase	Sponsor/company	NCT identifier
APH-1105	• Dementia	Intranasal nanoparticles	2	Aphios Pharma LLC, United Kingdom	NCT03806478
	• Alzheimer’s disease				
JNJ-39393406	• Schizophrenia	Nanosuspension	1	Janssen Pharmaceutica N.V., Belgium	NCT01137799
	• Alzheimer’s disease				
Stem cell treatment	• Alzheimer’s dementia	Intravenous/intranasal topical bone marrow stem cell (BMSC) fraction	Not applicable	MD Stem Cells Westport, Connecticut, United States	NCT03724136
	• Lewy Body Dementia				
	• Huntington’s dementia				
	• Traumatic brain injury				
Combination of intranasal insulin and empagliflozin	• Cognitive impairment	Aptar Pharma CPS Intranasal Delivery Device	Phase 2	Wake Forest University Health Sciences	NCT05081219
	• Alzheimer’s disease				
Syntocinon	• Frontotemporal dementia	Nasal spray	Phase 2	UCLA Los Angeles, California, United States	NCT03260920
Tdap-combined vaccine	• Alzheimer’s disease	Nasal vaccines	Phase 1	Mindful Diagnostics and Therapeutics, LLC	NCT05183516
			Phase 2		

## 4 Conclusion

Neurodegenerative disorders such as Alzheimer’s disease (AD), migraine, schizophrenia, Parkinson’s disease, and brain injury lead to memory impairments. Physiological barriers like blood–brain barrier (BBB), blood–cerebrospinal fluid barrier along with efflux transporters like P-glycoprotein (P-gp) prevents effective therapy using neurotherapeutics. Conventional oral routes offer mere superficial therapy, while invasive delivery mechanism leads to adverse cardiac and extrapyramidal side effects. The intranasal route of administration has, thus, surfaced as a valuable alternative with noninvasive, effective, safe, and targeted delivery of neurotherapeutics. Additionally, nanocarriers help achieve better brain targeting *via* the olfactory nerve pathway and trigeminal nerve pathway of the nasal cavity, therein bypassing the brain barriers and improving the diffusion of drug molecules. However, this promising combination of nanocarriers and intranasal delivery needs to elucidate better clinical profiles, pharmacodynamics, and pharmacokinetics for sustainable recovery from neurodegenerative diseases and complications.

## Abbreviations

AD, Alzheimer’s disease; NFTs, neurofibrillary tangles; FDA, Food and Drug Administration; BBB, blood–brain barrier; CNS, central nervous system; SLNs, solid lipid nanoparticles; NLC, nanostructured lipid carrier; WHO, World Health Organization; LDCs, lipid drug conjugates; ME, micro emulsion; mfsd2a, major facilitator superfamily domain-containing protein 2A; NE, nanoemulsion; P-gp, P-glycoprotein; AChEIs, acetyl cholinesterase inhibitors; CNT, carbon nanotubes; MWCNT, multi-walled carbon nanotubes.
